# Lipid Target in Very High-Risk Cardiovascular Patients: Lesson from PCSK9 Monoclonal Antibodies

**DOI:** 10.3390/diseases6010022

**Published:** 2018-03-17

**Authors:** Giovanni Ciccarelli, Saverio D’Elia, Michele De Paulis, Paolo Golino, Giovanni Cimmino

**Affiliations:** Department of Cardio-Thoracic and Respiratory Sciences, Section of Cardiology, University of Campania “Luigi Vanvitelli”, 80131 Naples, Italy; ciccarelli.giovanni@gmail.com (G.C.); saveriodelia85@gmail.com (S.D.); mdepaulis89@gmail.com (M.D.P.); paolo.golino@unicampania.it (P.G.)

**Keywords:** lipoproteins, atherosclerosis, cardiovascular risk, statin, PCSK9

## Abstract

The role of low-density lipoproteins (LDLs) as a major risk factor for cardiovascular disease has been demonstrated by several epidemiological studies. The molecular basis for LDLs in atherosclerotic plaque formation and progression is not completely unraveled yet. Pharmacological modulation of plasma LDL-C concentrations and randomized clinical trials addressing the impact of lipid-lowering interventions on cardiovascular outcome have clearly shown that reducing plasma LDL-C concentrations results in a significant decrease in major cardiovascular events. For many years, statins have represented the most powerful pharmacological agents available to lower plasma LDL-C concentrations. In clinical trials, it has been shown that the greater the reduction in plasma LDL-C concentrations, the lower the rate of major cardiovascular events, especially in high-risk patients, because of multiple risk factors and recurrent events. However, in a substantial number of patients, the recommended LDL target is difficult to achieve because of different factors: genetic background (familial hypercholesterolemia), side effects (statin intolerance), or high baseline plasma LDL-C concentrations. In the last decade, our understanding of the molecular mechanisms involved in LDL metabolism has progressed significantly and the key role of proprotein convertase subtilisin/kexin type 9 (PCSK9) has emerged. This protein is an enzyme able to bind the LDL receptors (LDL-R) on hepatocytes, favoring their degradation. Blocking PCSK9 represents an intriguing new therapeutic approach to decrease plasma LDL-C concentrations, which in recent studies has been demonstrated to also result in a significant reduction in major cardiovascular events.

## 1. Introduction

A strong correlation between lipid plasma levels and atherosclerotic cardiovascular disease (ASCVD) has been clearly shown over the years [1]. Lipid deposition, mainly low-density lipoprotein (LDL), occurs in the arterial wall of almost all vascular districts, defining the atherosclerotic process, with focal clinical manifestations based on the organ damaged: heart (coronary artery disease), brain (cerebrovascular disease), and/or limbs (peripheral vascular disease) [2]. Plaque complication results in exposure of prothrombotic material to the flowing blood, leading to acute thrombus formation, which may result in an acute medical emergency, such as acute coronary syndrome (ACS), stroke, or related problems [3,4,5]. Lowering plasma LDL concentrations is highly recommended for patients with hyperlipidemia and multiple risk factors, such as diabetes, hypertension, smoking status, chronic kidney disease, or peripheral artery disease [6,7]. According to the current guidelines, diet and weight loss represent the first “medical” approach [1,8]. However, pharmacological modulation of plasma lipid concentrations is quite often necessary according to the target lipid levels.

The majority of clinical trials have shown the efficacy of statins in reducing major cardiovascular events (MACE) in both primary and secondary prevention [9,10]. Moreover, because of their low cost, they are sustainable from the economic point of view. However, despite the proven efficacy, in a subset of patients achievement of the plasma cholesterol target by statins remains an unmet clinical need because of: (1) intolerance (defined as muscle pain and/or liver dysfunction) [11]; (2) not powerful enough to achieve the target [12]; (3) unfavorable genetic background, (i.e., heterozygous familial hypercholesterolemia (FH) or homozygous FH [13]).

In the last 15 years, new insight into the basic mechanisms involved in cholesterol metabolism has been gained, and new pharmacological targets have been identified [14]. The proprotein convertase subtilisin/kexin type 9 (PCSK9) is an example of rapid pre-clinical and clinical progression between their discovery in 2003 and the present because of the potential the innovative therapeutic scenario opened up [15]. This protein is a key player in the clearance of LDL particles and its inhibition seems to be highly effective in reducing plasma LDL-C concentrations [16]. This review, starting with the role of cholesterol in the pathogenesis of atherosclerosis in high-risk patients, will discuss the novelty of the PCSK9 approach and the available data on the safety and effectiveness of its inhibition in reducing plasma LDL-C concentrations.

## 2. Traditional Pharmacological Approaches for Dyslipidaemias: From Bench to Bedside

### 2.1. Atherosclerotic High-Risk Patients

Atherosclerosis is a multifactorial, chronic “inflammatory-degenerative” disease of the arterial tree [3,17], recognizing in the atheroma its pathological substrate [18]. Conventional (hypertension, diabetes, smoking, hyperlipidemia, age, sex) and less conventional (impaired glucose tolerance; impaired fasting glucose, apolipoprotein B (ApoB); apolipoprotein A-I (ApoAI), triglycerides; triglyceride-rich lipoproteins[TGRLs], small and dense LDL, oxidized-LDL, antibodies against oxidized-LDL, Lipoprotein (a), homocysteine, high-sensitivity C-reactive protein) cardiovascular risk factors are the main determinants for the impairment of the endothelial protective properties [19]. The resulting endothelial dysfunction [20,21,22,23] with deposition of circulating LDL-C in the arterial wall is the initial step of the atherosclerotic process [24]. Within the subendothelial space, LDL-C become more susceptible to oxidation due to local reactive oxygen species (ROS) production, triggering the atherosclerotic cascade that leads to plaque formation, progression, and destabilization [2]. In each step of this process, the role of inflammation and immunity is now well defined [3,25].

Patients with diabetes mellitus (DM) are considered at high cardiovascular risk, with a ~10-fold increased risk in their lifetime [26,27,28]. Increased plaque vulnerability [29], advanced glycosylation end products (AGEs) formation [30,31], enzyme-mediated endothelial damage [30], and a modified lipid profile (with an increase in VLDLs and “small and dense” LDLs) [32] indicates that diabetic patients may have accelerated atherosclerosis.

Patients with chronic kidney disease (CKD) also show an increased risk for ASCVD [33], mainly related to a loss of renal parenchyma, which accelerates atherosclerosis [34], a chronic inflammation status with elevated CRP levels, and reduced renal clearance of several cytokines, such as IL-1β, IL-6, and TNF-α [35], as well as raised levels of angiotensin II and parathormone, which contribute to increased ROS production and calcium deposition in the vessel wall, thus inducing endothelial dysfunction [33]. Coronary plaques from CKD patients show extensive calcification and increased presence of thrombotic events [36].

Autopsy studies have shown that fibrous plaques are more common in the femoral arteries [37,38]. Peripheral atherosclerotic disease occurs in the context of multiple disease processes that interfere with exercise ability. Potential mechanisms include the conventional risk factors in a local setting of reduced blood flow, altered muscle metabolism, and impaired angiogenesis, leading to limb discomfort and functional limitation [39].

### 2.2. Major Drugs Used in LDL Cholesterol-Lowering Strategies

Several randomized clinical trials have unequivocally shown that lipid-lowering therapies are associated with MACE reduction [40,41,42]. Despite dietary approach, physical activity, and weight loss being the strategies recommended first [43], many pharmacological strategies have been identified to modulate plasma lipid levels.

(a) *Statins:* Inhibition of HMG-CoA reductase by statins has been the first therapeutic approach. These drugs block the endogenous synthesis of cholesterol in the liver, resulting in a reduction of intracellular cholesterol. This causes induction of LDL-R expression of the hepatocyte surface, which in turn leads to enhanced clearance of LDL particles from the blood. Several trials have highlighted an improvement in terms of cardiovascular morbidity and mortality as well as the need for coronary artery interventions using statins [44,45]. This protective effect is of greater magnitude (a) if statin treatment is started earlier and lasts a longer time; (b) if a high dose is given [9,10,46,47]; and/or (c) if the percentage LDL-C reduction from baseline value is higher [48]. The PROVE IT-TIMI 22 trial (Pravastatin or Atorvastatin Evaluation and Infection Therapy–Thrombolysis in Myocardial Infarction 22) demonstrated that plasma LDL-C concentrations at baseline are an important predictor of the benefit of intensive lipid-lowering therapy [48]. Indeed, decreasing LDL-cholesterol baseline levels is an additional benefit of intensive treatment with statins compared with moderate-dose therapy declines. Consequently, current guidelines suggest that HMG-CoA reductase inhibitors represent the first choice for patients with hypercholesterolemia or combined hyperlipidemia [1].

(b) *Selective cholesterol absorption inhibitors:* By inhibiting intestinal cholesterol and phytosterol absorption protein (Niemann-Pick C1-Like 1, NPC1L1) present on jejunal intestinal cells, ezetimibe decreases intestinal cholesterol absorption from dietary sources and from bile, resulting in a reduction in hepatic cholesterol concentration and circulating LDL-C by up to 20% [14]. The IMPROVE-IT trial (Improved Reduction of Outcomes: Vytorin Efficacy International Trial) concluded that the combination of ezetimibe plus simvastatin as compared to simvastatin alone in patients with acute coronary syndromes resulted in a further lowering of plasma LDL-C concentrations by up to 50 mg/dL, with an associated improvement in cardiovascular outcomes [49].

(c) *Fibrates:* Fibric acid derivatives, or fibrates, are agonists of α isoform of peroxisome proliferator-activated receptor (PPAR). Activation of this receptor may result in several modifications of the plasma lipid profile [50]. Five major effects have been characterized by the use of fibrates: (1) Lipoprotein lipolysis induction via lipoprotein lipase activity and apoC-III inhibition; (2) increased hepatic fatty acid uptake and reduction of hepatic triglyceride production (these two effects result in hypotriglyceridemic action); (3) formation of LDL particles with a higher affinity for the own receptor, thus resulting in a higher rate of LDL particles removal; (4) reduction in cholesteryl ester and triglycerides exchange between VLDL and HDL, leading to decreased plasma levels of triglyceride-rich lipoproteins; and (5) increase in the production of apoA-I and apoA-II in liver, thus inducing a higher HDL production and promoting reverse cholesterol transport [51,52]. However, because of the variable results from the clinical trials investigating the impact of fibrates on clinical outcomes, either in primary and secondary prevention, and problems linked to safety, their role remains limited to selected patients with diabetes, metabolic syndrome, or dyslipidemia [53,54,55,56].

A large amount of clinical trial data indicate that the central point of all lipid-lowering strategies for high-risk patients is the percentage change from baseline rather than any predefined target. This view is corroborated by the following findings: (1) on-treatment LDL-C plasma concentrations levels do not predict CVD risk rates, whereas baseline LDL-C plasma concentrations do; (2) the correlation between LDL reduction and CVD risk reduction within each study is at best curvilinear even in high-risk, high-cholesterol populations where the average on-treatment LDL is still significantly distant from the predefined target of 100 mg/dL; (3) despite the same percentage of LDL reduction, more benefits have been reported in subjects with higher baseline LDL (and whose on-treatment LDL stays higher than 100 mg/dL). Despite the achieved LDL-C goal, in very high-risk patients recurrent events might occur. By taking into account the linear correlation between LDL-C level reduction and the risk of fatal or nonfatal MACE, a further percentage variation from their baseline (even if it is low) might result in additional benefits [57]. PCSK9 inhibition by monoclonal antibodies opens up a new way to achieve this goal.

## 3. PCSK9 Inhibition: A Route to Very Low LDL-C Plasma Concentrations

Because of a residual cardiovascular risk in patients not at the target LDL level or with a percentage variation from baseline still low, researchers are still looking for the best pharmacological strategy.

The discovery of proprotein convertase subtilisin/kexin type 9 (PCSK9) has created a new frontier for better management of dyslipidemia. Indeed, in 2003 Abifadel et al., studying gene mutations responsible for familial hypercholesterolemia (FH), found that mutations in the *PCSK9* gene cause dominant hypercholesterolemia in pedigree analysis. Interestingly, a gain of function (GOF) due to a missense mutation in this gene was the cause of the disease [58]. To date, three gene mutations are known to cause FH: (1) the LDL receptor itself (FH1); (2) apolipoprotein (apo) B (FH2), a ligand of LDL receptors; and (3) a GOF mutation in PCSK9 (FH3).

**Figure 1:**
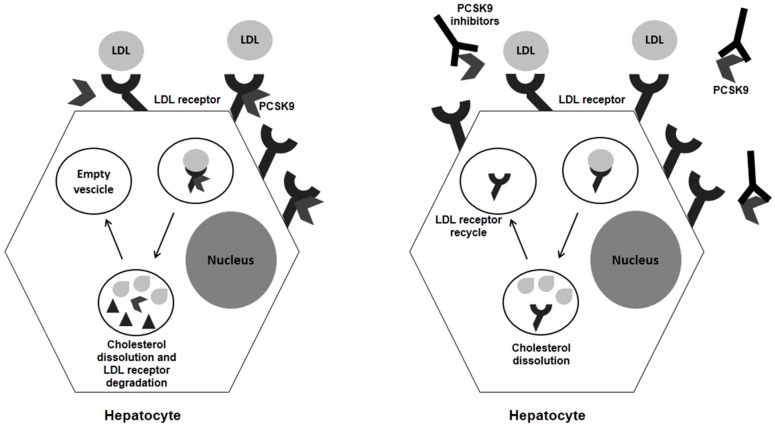
Schematic view of PCSK9 activity and effects of its inhibition.

A better understanding of the PCSK9 pathway showed that this molecule promotes the degradation of LDL receptors by forming an enzyme–substrate complex, mainly in the liver. The cell-surface complex LDL-R/LDL is transported to the endosomes via endocytosis, where LDLs are released in acid conditions. Within the endosome, LDL is degraded to amino acids and cholesterol while LDL-R is transported back to the cell surface, but, if bound to PCSK9, LDL-R will be degraded too (Figure 1). Therefore, PCSK9 inhibition may be an effective strategy to promote LDL-R recycling, thus reducing circulating LDL particles (Figure 1). The rate of plasma LDL-C decrease is approximately 45–60%, whether used alone or in combination with a statin. Addition of an anti-PCSK9 antibody to standard therapy—with statin alone, or statin combined with Ezetimibe—resulted in a further reduction of plasma LDL-C concentrations (up to 60%) and a halved cardiovascular event rate compared to the placebo [59].

Evolocumab and Alirocumab are “fully” humanized anti-PCSK9 antibodies, while Bococizumab is a humanized monoclonal antibody. These three monoclonal antibodies are currently under investigation in extensive clinical programs and trials, namely PROFICIO, ODYSSEY, and SPIRE. The latest was discontinued in November 2016.

As reported above, the PROFICIO (Program to Reduce LDL-C and Cardiovascular Outcomes Following Inhibition of PCSK9 in Different Populations) is the development program for Evolocumab, a monoclonal antibody already approved by the FDA and EMA. Several trials of this program have already reported efficacy (with a mean of LDL-C reduction up to 57%), safety, and durable effects in different populations [14,60,61], even patients affected by LDL receptor abnormalities such as homozygous or heterozygous Familial Hypercholesterolemia (FH) [62,63]. The first direct effect of PCSK9 inhibition by evolocumab on atherosclerotic plaque comes from the GLAGOV study, published in 2016 [64]. Coronary atherosclerotic lesions have been evaluated by intravascular ultrasound in patients receiving evolocumab or placebo on top of statin therapy. Among patients with angiographic evidence of coronary artery disease and on chronic statin therapy, the PCSK9 inhibitor evolocumab resulted in a greater change in atheroma volume (−0.95% vs. +0.05% of the placebo group) and a greater proportion of patients with plaque regression with a mean plasma LDL-C concentrations in the active drug group of 36.6 mg/dL [64]. Moreover, the latest results available from the FOURIER study, a large-scale outcome study in 27,564 ASCVD patients on statin therapy presented in 2017, showed that, with the addition of evolocumab, the mean plasma LDL-C concentrations dropped to 30 mg/dL. In addition, in a median follow-up period of 2.2 years, there was a decrease in the rate of the composite primary endpoint of cardiovascular death, myocardial infarction, stroke, hospitalization due to unstable angina, and coronary artery revascularization (percutaneous coronary intervention, coronary artery bypass graft) of 15% compared with the placebo [65].

Another big program is the ODYSSEY trials testing Alirocumab. As for evolocumab, several data from this program have already been published, showing the power and safety of this approach in reducing LDL-C plasma concentrations by up to 47% in patients at high risk [14,60,66]. The latest results from the ODYSSEY Outcomes trial, presented at the American College of Cardiology in 2018, indicate that treatment with alirocumab reduced cardiovascular outcomes and all-cause deaths by 15% in a group of high-risk patients. These data reinforce the previous data from the FOURIER trial and expand them because of a longer outcome and a higher-risk population. A mean of 53.3 mg/dL of LDL-C plasma concentration was achieved in the alirocumab group, with a percentage variation of 54.7. The benefits were more marked in those patients with the highest LDL cholesterol at baseline. In the pre-specified post hoc analysis by LDL-C level at baseline, patients with an LDL-C ≥100 mg/dL experienced reductions in all endpoints. In the alirocumab group a 24% reduction in MACE was reported with an absolute risk reduction (ARR) of 3.4%. In detail, CHD death was reduced by 28% (ARR 0.9%), CV death by 31% (ARR 1.3%), and all-cause death by 28% (ARR 1.7%).

The FOURIER and the ODYSSEY Outcomes trials consistently show benefits from PCSK9 inhibitors, not only in terms of preventing nonfatal events such as heart attacks but in actually preserving life. Thus, the future is now.

A major warning raised by some recent data exploring the impact of PCSK9 inhibitors in cardiovascular outcome, was the possible higher rate of neurocognitive adverse events [61]. However, more recent analysis seems to not confirm this correlation. In two pre-specified analyses of the FOURIER study, despite slightly more injection-site reactions in the evolocumab arm, the clinical efficacy and safety of the PCSK9 therapy have been confirmed [67], with no difference between the groups especially regarding cognitive impairment (EBBINGHAUSS study) [68] and new-onset diabetes [69].

Moreover, the beneficial effects of lipid-lowering therapy with a non-statin agent added to high- or moderate-intensity statin therapy have also been reported in patients with symptomatic lower extremity peripheral artery disease (PAD), including those without prior MI or stroke [70].

Finally, a more recent meta-analysis found evidence of a significantly greater reduction of plasma LDL-C concentrations in patients treated with evolocumab than those treated with alirocumab [66].

The third monoclonal PCSK9 inhibitor antibody, namely Bococizumab, was discontinued in November 2016 because of neutralizing antibodies [71]. However, its effects have been described in two large-scale cardiovascular outcomes trials, SPIRE-1 and SPIRE-2, where a total of 27,438 participants with either a history of cardiovascular disease or familial hypercholesterolemia or high risk for cardiovascular disease were randomized to either bococizumab subcutaneously or a matching placebo. Despite the discontinuation, the results from both SPIRE trials confirmed the efficacy of PCSK9 inhibition in terms of very low plasma LDL-C concentrations and cardiovascular event reduction [72].

At the time of this review, new pharmacological approaches are under development to safely inhibit PCSK9 (Table 1). The latest strategy involves the use of a small interfering RNA (siRNA) [73]. The siRNA molecules, namely inclisiran, engage the natural pathway of RNA interference (RNAi) by binding intracellularly to the RNA-induced silencing complex (RISC), enabling it to cleave messenger RNA (mRNA) molecules specifically encoding PCSK9. The cleaved mRNA is degraded and thus unavailable for protein translation, which results in decreased levels of the PCSK9 protein [74].

Based on the current available evidence, a PCSK9 inhibitor should be considered in specific subsets of patients:

- Patients with ASCVD at very high risk of an adverse prognosis, with persistent elevated plasma LDL-C concentrations despite maximally tolerated statin alone or in combination with ezetimibe therapy.
- Patients with ASCVD at very high risk with persistent elevated plasma LDL-C concentrations, who show intolerance to the appropriate doses of at least three statins.
- Familial hypercholesterolemia patients without clinically diagnosed ASCVD, at high cardiovascular risk, with persistent elevated plasma LDL-C concentrations despite maximally tolerated statin plus ezetimibe therapy [75].

## 4. Metabolic Effects of PCSK9 Inhibition: Beyond LDL-C Reduction

In humans, PCSK9 expression has been reported in different organs (i.e., brain, kidney, pancreas, liver, and small intestine) [76] and cells (i.e., endothelium, smooth muscle cells, and macrophages) [77,78], suggesting systemic and local homeostatic effects. Based on these data, blocking PCSK9 may result in unexpected effects. 

### 4.1. PCSK9 and Glucose Metabolism

It is known that perturbation of cholesterol metabolism may expose patients to increased risk of diabetes, since statin treatment [79] and genetic variant of HMGCoA reductase [80] have been associated with a higher prevalence of type 2 diabetes. Moreover, statin use and genetic inhibition of HMGCoA reductase are associated with increased body weight [80,81,82], thus resulting in metabolic syndrome and diabetes predisposition. These data seem to be confirmed by the evidence that patients with familial hypercholesterolemia showed a lower prevalence of glucose metabolism impairment [83].

Inhibition of PCSK9 in pancreatic cells decreases cholesterol accumulation, which results in glucose metabolism impairment and reduction of insulin secretion, thus favoring diabetes status [84]. However, contradictory data are available on this issue, since PCSK9 loss of function (LOF) is not associated with increased risk of diabetes [85] while the PCSK9 R46L variant seems to be associated with insulin resistance [86]. From a metabolic point of view, PCSK9, by decreasing LDL-R activity, reduces cholesterol concentrations within the pancreatic beta-cell, finally resulting in increased beta-cell function and insulin secretion. Moreover, the direct effect of PCSK9 on pancreatic delta-cell leads to increased insulin secretion, thus resulting in beneficial effects on carbohydrate homeostasis. On the other hand, PCSK9 induces insulin-regulated secretion via SREBP-1C with increased glucose, HbA1c, and HOMA-IR index, leading to a detrimental effect on carbohydrate homeostasis, thus counterbalancing the beneficial effect of the other metabolic pathway. Based on these data, PCSK9 inhibition should result in a neutral effect [84].

The latest analysis of the published clinical trials, looking specifically at the new onset of diabetes, seems to support this neutral effect [67,69]. However, the relatively short time of observation of these studies may represent a major limitation. More recently, Cao et al. [87] published a systematic review and meta-analysis of a total of 18 studies including 26,123 participants treated with alirocumab or evolocumab and without diabetes. PCSK9 inhibition had no significant impact on new-onset diabetes mellitus and glucose homeostasis, regardless of PCSK9-mAb type, participant characteristics, treatment duration, treatment method, and differences in control treatment, thus adding new evidence for the neutral effect of PCSK9 on glycemic control.

### 4.2. PCSK9 and Lipogenesis

PCSK9 not only targets LDL-R but may regulate other steps and key players in lipogenesis [76]. Recently, Verbeek et al. [88] have analyzed the lipid profile and lipoprotein subfractions by nuclear magnetic resonance in carriers of R46L variant that are responsible for a PCSK9 LOF. The population enrolled was part of the European Prospective Investigation into Cancer and Nutrition (EPIC) study, designed to investigate the relationships between diet, nutritional status, lifestyle, environmental factors, and the incidence of cancer and other chronic diseases. The data from this analysis confirm and expand previous results [89] on the effects on other lipoproteins (such as Apolipoprotein B (ApoB), Lp (a), secretory phospholipase A2, and Lp-PLA2( beyond the already known decrease in LDL-C plasma concentrations. Specifically, IDL and VLDL particles were subject to a variation of −18% and −16%, respectively. No effect on HDL-C plasma concentrations has been reported [88]. These observations may indicate that the positive effects of PCSK9 inhibition, especially on patients at high risk, may be related, at least in part, to the reduction of most atherogenic lipoproteins, lifelong exposure to which is associated with an increased cardiovascular risk. It has been reported that PCSK9 circulates in the flowing blood as lipoprotein-bound, mainly associated with ApoB within LDL and VLDL particles, thus suggesting an involvement in another molecular pathway beyond the LDL. Confounding data are available at the time of this article on the interaction between PCSK9 and VLDL-R, ApoE-R2, and CD36. Because these receptors are involved in the basal and post-prandial plasma triglycerides levels, PCSK9 may be indirectly involved in this pathway [90]. A gene silencing study with siRNA and clinical observations in carriers of GOF and LOF variants have been performed.

Compared to patients carrying an LDL-R mutation, individuals with a PCSK9 GOF variant show higher levels of VLDL, IDL, and triglycerides beyond the increased LDL-C plasma concentrations. On the other hand, individuals with a PCSK9 LOF variant, especially the R46L, show a reduction in all ApoB-containing lipoproteins [90], and attenuated levels of fasting and postprandial triglycerides [91]. Based on these observations, a putative role of PCSK9 in the clearance of triglyceride-rich lipoproteins (TGRLs) may be postulated. Similar results have been obtained by inhibiting PCSK9 via gene silencing or viral transfection, thus suggesting a non-direct effect of PCSK9 on triglyceride metabolism [90]. Moreover, a recent study confirms that Alirocumab reduces ApoB levels in both IDL and LDL, most likely due to increased LDL degradation [92]. It is known that ApoB turnover is strongly influenced by the uptake of lipoproteins containing ApoB, mediated by LDL-R [93]. Results from the FOURIER trials indicate that PCSK9 inhibition is associated with a mild–moderate reduction in triglyceride levels [59]. The biological mechanism underlying this effect may include several pathways beyond the increased LDL-R activity that are associated with the increased catabolism of TGRLs [94]. It is known that PCSK9 modulates lipoprotein assembly and secretion by the intestine and the liver and affects TGRL and fatty acid uptake in peripheral tissues via expression of the very-low-density lipoprotein receptor (VLDL-R), the ApoE2 receptor, and the CD36 receptor [16,76]. The receptor for VLDL modulates the extra-hepatic metabolism of TGRLs in concert with lipoprotein lipase, thus contributing to the delivery of fatty acids to these peripheral tissues, especially in the heart, skeletal muscle, and adipose tissue, where it is highly expressed.

However, despite the reduction in triglycerides (up to 17.3%), ApoB (up to 56%) and the positive outcomes in the clinical trials testing PCSK9 inhibition by both monoclonal antibodies, alirocumab and evolocumab, the clinical impact of the non-LDL cholesterol concentrations on CV risk remain unclear.

Summarizing, the lower plasma triglyceride concentrations and the decreased postprandial lipemia may be the results of the following mechanisms [94]: (a) reduced intestine ApoB48 production, leading to decreased chylomicron secretion; (b) increased ApoB degradation, resulting in reduced ApoB-rich lipoproteins; (c) increased chylomicrons, chylomicron remnants, and VLDL remnants clearance via increased LDL-related protein 1 activity and CD36 scavenger receptor upregulation; (d) VLDL receptors and ApoE receptors.

### 4.3. PCSK9 and HDL Particles

Finally, the available clinical trials (OSLER [95], ODYSSEY Long Term [96], and FOURIER [65]) indicate that PCSK9 inhibition may also affect HDL metabolism. A modest increase in plasma HDL-C and apoA1 concentrations (estimated at less than 10%) has been reported. This effect may be the result of two potential mechanisms: (a) the reduced number of LDL particles leads to a decreased transfer of cholesterol from HDL to LDL particles [97,98]; (b) the blockage of PCSK9 reduces cholesterol ester transfer protein (CETP) activity, thus decreasing heteroexchange of lipids between TGRLs and HDL particles [99].

## 5. “Very Low Is Better”: End of Story?

The progression of pharmacological modulation of lipid metabolism and the available data on safety and efficacy of the current strategies indicate that more ambitious targets in terms of lipid lowering can be achieved. Randomized clinical trials published to date clearly showed that PCSK9 inhibitors may result in plasma LDL-C concentrations lower than 50 mg/dL easily with no safety concern [100]. More interestingly, a recent report from Ference et al. quantified the reduction of cardiovascular events in about 20% per decrease of 1.00 mmol/L (39 mg/dL) in plasma LDL-C concentrations [101]. These effects mediated by PCSK9 inhibitors are independent and additive with statins. Thus, taking into account the fact that inhibition of PCSK9 results in a strong reduction of plasma LDL-C concentrations, the benefit expected will be higher, especially in patients with an elevated baseline value.

Based on these results, the latest guidelines from the American Association of Clinical Endocrinologists and American College of Endocrinology [102] identify an “extreme risk” group (Table 2a), beyond the very high-risk group already defined in the European Society of Cardiology Guidelines 2016 [103] (Table 2b), characterized by progressive atherosclerotic cardiovascular disease, including unstable angina that persists after achieving an LDL-C less than 70 mg/dL, or established clinical ASCVD with diabetes, stage 3 or 4 CKD, and/or HeFH, or in those with a history of premature ASCVD (<55 years of age for males or <65 years of age for females) in which a LDL-C goal of less than 55 mg/dL is recommended. The goal of 30 mg/dL, as indicated in most of the PCSK9 inhibitors clinical trials, is desirable.

However, how safe it is to go “very low” remains to be elucidated [104] and will be a matter for the long-term outcomes study to determine in the next few years. On this safety concern, some epidemiological studies available to date indicate a different association between cholesterol plasma concentration and neoplastic risk, hemorrhagic stroke, depression and anxiety, and nervous and immune system dysfunction. Specifically, decreased plasma LDL-C concentrations have been associated with increased cancer risk [105]. In a study from Benn et al. including participants from the Copenhagen City Heart Study and the Copenhagen General Population Study, low plasma LDL-C concentrations were firmly associated with increased cancer risk, but genetically decreased LDL-C was not [106]. Whether this association is causal remains unclear. Low LDL-C plasma concentrations may be a consequence, rather than a cause, of the neoplastic disease, thus may not cause cancer per se. Pre-specified clinical trials designed to address this safety issue are warranted.

## Figures and Tables

**Table 1:** PCSK9 under development.

Drugs	Type	Status	Study
Evolocumab	Monoclonal Ab	Approved	Proficio Program
Alirocumab	Monoclonal Ab	Approved	Odyssey Program
Bococizumab	Monoclonal Ab	Discontinued	Spire Program
Inclisiran	Silent RNA	On approval	Orion 1
LGT-209	Monoclonal Ab	Discontinued	-
RG7652	Monoclonal Ab	Phase 2	Equator
ALN-PC	RNAinhibitor	Phase 1 ev/Preclinical sc	-
Adnectin BMS-962476	modified binding protein	Phase 1	-
EGF-A peptide	synthetic peptide	Preclinical	-

**Table 2:** Current recommendations.

a. **Recommendations of AACE 2017 (American Association of Clinical Endocrinologists and American College of Endocrinology)**			**Achieved LDL-C by PCSK9 mAb [60]**
**EXTREME risk:** Progressive atherosclerotic cardiovascular disease, including unstable angina that persists after achieving an LDL-C less than 70 mg/dL, or established clinical ASCVD with diabetes, stage 3 or 4 CKD, and/or HeFH, or in those with a history of premature ASCVD (<55 years of age for males or <65 years of age for females)	LDL-C goal of less than 55 mg/dL is recommended	Grade A; BEL 1	**−43.8 to −55.2%** in HeHF with baseline value ≥100 mg/dL **48 mg/dL** in high ASCVD
**VERY HIGH risk:** Established or recent hospitalization for ACS; coronary, carotid or peripheral vascular disease; diabetes or stage 3 or 4 CKD with one or more risk factors; a calculated 10-year risk greater than 20%; or HeFH	LDL-C goal of less than 70 mg/dL is recommended	Grade A; BEL 1	**−36.3 to −54%** In patients with prior CV disease + LDL-C ≥ 70 mg/dL,
**HIGH risk:** An ASCVD equivalent including diabetes or stage 3 or 4 CKD with no other risk factors, or individuals with two or more risk factors and a 10-year risk of 10–20%	LDL-C goal of less than 100 mg/dL is recommended	Grade A; BEL 1	**−36.3 to −54%** In patients with CV risk factors + LDL-C ≥ 100 mg/dL
**MODERATE risk:** Two or fewer risk factors and a calculated 10-year risk of less than 10%	LDL-C goal of less than 100 mg/dL is recommended	Grade A; BEL 1	**−58.7%** Patients not having adequate control of their hypercholesterolemia based on their individual level of CVD risk
**LOW risk: No risk factors**	For individuals at low risk (i.e., with no risk factors), an LDL-C goal of less than 130 mg/dL is recommended.	Grade A; BEL 1	
b. **Recommendations of ESC (European Society of Cardiology) Guidelines 2016**			
**VERY HIGH CV risk:** -Documented CVD -DM or type-1 DM with TOG -Severe RD: GFR <30 mg/mL/1.73 m^2^ −10-year risk SCORE ≥10%	**VERY-HIGH CV risk:** LDL-c goal <70 mg/dL (1.8 mmol/L) and/or 50% reduction if baseline is 70–135 mg/dL (1.8–3.5 mmol/L)	CLASS I LEVEL B	
**HIGH CV risk:** -Markedly elevated single risk factor −10-year risk SCORE ≥5% and <10% -Moderate RD: GFR 30–59 mg/mL/1.73 m^2^	**HIGH CV risk:** LDL-c goal <100 mg/L (2.6 mmol/L) or 50% reduction if baseline is 100–200 mg/dL (2.6–5.1 mmol/L)	CLASS I LEVEL B	
**MODERATE CV risk:** −10-year risk SCORE ≥1% and <5%	**MODERATE CV risk:** LDL-c goal <115 mg/dL (3.0 mmol/L)	CLASS IIa LEVEL C	
**LOW CV risk:** −10-year risk SCORE <1%			
